# Significance of Antioxidants on Aging and Neurodegeneration

**DOI:** 10.3390/ijms232213957

**Published:** 2022-11-12

**Authors:** Jana Tchekalarova, Rumiana Tzoneva

**Affiliations:** 1 Institute of Neurobiology, Bulgarian Academy of Sciences, Acad. G. Bonchev Street, Block 23, 1113 Sofia, Bulgaria; 2 Institute of Biophysics and Biomedical Engineering, Bulgarian Academy of Sciences, Acad. G. Bonchev Street, Block 21, 1113 Sofia, Bulgaria

The hallmark of aging is an organism’s difficulty to maintain proper homeostasis, leading to a disrupted balance between the endogenous antioxidant system and the production of free radicals, a progressive inflammatory process, and increased susceptibility to (neurodegenerative diseases. The most common age-related neurodegenerative diseases, such as Alzheimer’s disease (AD), Parkinson’s disease (PD), Huntington’s disease, and Epilepsy have a complex molecular origin with multiple etiologies. Furthermore, increased oxidative stress is a common factor in the progression of these diseases. In recent years, therapeutic strategies that use antioxidants to delay the progression of aging and concomitant neurodegenerative processes have been developing in several ways.

It has been suggested that aging could be provoked by a disturbed prooxidant/antioxidants ratio, leading to oxidative stress [1]. While the enhanced production of free oxygen radicals is a typical process in cellular metabolism, their accumulation in the body due to the impaired mechanism of their clearance is a prerequisite for pathogenesis and diseases. Brain tissue is one of the most vulnerable tissues to oxidative stress is because of its elevated levels of polyunsaturated fatty acids, iron, and high oxygen saturation [2].

In this Special Issue, various topics on the essential impact of antioxidants on aging and neurodegeneration are described, including the pathological condition in either aging or a model of neurodegenerative disease and possible therapeutic options. The disturbed clearance of free radicals, such as the superoxide radical, hydroxyl radical, and singlet oxygen, demonstrated that, after 24 h, the pilocarpine-induced status epilepticus in the rat hippocampus increased lipid peroxidation and blunted the activity of endogenous antioxidants, SOD and GSH [3]. The study by Shishmanova et al. [3], published in this Special Issue, reported that the antioxidant effect of a third-generation antiepileptic drug (AED) Lacosamide (LCM), used for patients with focal epilepsy, and the second-generation AED topiramate (TPM), applied for a broad range of seizures, is an essential mechanism of its seizure activity suppression and its deliberating consequences. The high dose of 30 mg/kg LCM elevated the activity of the SOD antioxidant enzyme, while both LCM and TPM restored the CAT activity and MDA levels in the hippocampus to control values in SE-induced rats.

The disturbed homeostasis associated with microglial activity and its impact on protein signaling cascades is also important in aging-related neuronal pathogenesis. In this Special Issue, Jalloh et al. [4] reported how treatment with polyphenols could benefit and delay age-related deleterious processes. They suggest that a month’s dietary supplementation of polyphenol NT-020 is effective for maintaining a more beneficial microglial phenotype during aging. Their findings will help to understand the impact of aging on the molecular mechanism of changes in rat microglia, providing a rational therapeutic approach for age-related microglial dysfunction.

The decline in cognitive capacity is a clinical symptom characteristic of aging and neurodegeneration diseases. It arises from the impairment of the hippocampus and cerebral cortex due to progressive neuroinflammation and activated glia (microglia and astrocytes). The therapeutic approaches to this delay in decreased cognitive function have been discussed for many years. Cichon et al. [5] reviewed the latest preclinical and clinical reports focused on the impact of antioxidants with natural origin on learning and memory, emphasizing age-associated neurodegenerative diseases. Many studies report that using a diet rich in antioxidants concomitant with reduced caloric intake may delay age-related memory impairment and reduce the risk of the onset of neurodegenerative diseases [6,7]. The authors of this review indicate some critical challenges that must be solved because of discrepancies between pre-clinical and clinical studies. Nevertheless, Cichon et al. [5] concluded that phytocompounds with antioxidant properties have advantages due to their low toxicity and side effects. These characteristics require continued research that uses more precise designs because they represent a promising alternative primarily as an adjuvant treatment for neurodegenerative diseases. 

The hormone melatonin, produced by the pineal gland, is considered a broad-spectrum antioxidant with a strong potential to neutralize free radicals and effectively protect cells. In this regard, Tchekalarova et al. [8] elucidated the role of the pineal gland in aging through the production of the hormone melatonin. Using an animal model of melatonin deficit in young adults, mature and aging rats, they demonstrated that pinealectomy had a crucial negative effect on emotional responses in young adult rats and provoked oxidative stress-induced changes in cholesterol and sphingomyelin (SM) levels in mature rats. 

Behl T. et al. [9] gave a brief contemporary overview of neurodegenerative diseases (NDs) and their associated molecular mechanisms, summarizing the popular nutraceuticals used as a a complementary therapy to starve NDs. Their attractive characteristics that aid in the fight against NDs are their natural source, lower toxicity, therapeutic interventions, biocompatibility, potential nutritional effects, and presence of various anti-oxidative and neuroprotective constituents, which act by altering multiple signaling pathways.

This Special Issue has an ambition to contribute to the understanding of the pathogenesis of both aging and neurodegenerative diseases with special emphasis on the role of oxidative stress and to shed light on the precise mechanism underlying the effects of antioxidants and key signaling pathways involved in neurodegeneration.

## References

[1] Junqueira V.B.C., Barros S.B.M., Chan S.S., Rodrigues L., Giavarotti L., Abud R.L., Deucher G.P. (2004). Aging and oxidative stress. Mol. Aspects Med..

[2] Méndez-Armenta M., Nava-Ruíz C., Juárez-Rebollar D., Rodríguez-Martínez E., Gómez P.Y. (2014). Oxidative stress associated with neuronal apoptosis in experimental models of epilepsy. Oxid. Med. Cell. Longev..

[3] Shishmanova-Doseva M., Peychev L., Yoanidu L., Uzunova Y., Atanasova M., Georgieva K., Tchekalarova J. (2021). Anticonvulsant Effects of Topiramate and Lacosamide on Pilocarpine-Induced Status Epilepticus in Rats: A Role of Reactive Oxygen Species and Inflammation. Int. J. Mol. Sci..

[4] Jalloh A., Flowers A., Hudson C., Chaput D., Guergues J., Stevens S.M., Bickford P.C. (2021). Polyphenol Supplementation Reverses Age-Related Changes in Microglial Signaling Cascades. Int. J. Mol. Sci..

[5] Cichon N., Dziedzic A., Gorniak L., Miller E., Bijak M., Starosta M., Saluk-Bijak J. (2021). Unusual Bioactive Compounds with Antioxidant Properties in Adjuvant Therapy Supporting Cognition Impairment in Age-Related Neurodegenerative Disorders. Int. J. Mol. Sci..

[6] Dias I.R., Santos C.d.S., e Magalhães C.O.D., de Oliveira L.R.S., Peixoto M.F.D., De Sousa R.A.L., Cassilhas R.C. (2020). Does calorie restriction improve cognition?. IBRO Rep..

[7] Madeo F., Carmona-Gutierrez D., Hofer S.J., Kroemer G. (2019). Caloric Restriction Mimetics against Age-Associated Disease: Targets, Mechanisms, and Therapeutic Potential. Cell Metab..

[8] Tchekalarova J., Nenchovska Z., Kortenska L., Uzunova V., Georgieva I., Tzoneva R. (2022). Impact of Melatonin Deficit on Emotional Status and Oxidative Stress-Induced Changes in Sphingomyelin and Cholesterol Level in Young Adult, Mature, and Aged Rats. Int. J. Mol. Sci..

[9] Behl T., Kaur G., Sehgal A., Singh S., Bhatia S., Al-Harrasi A., Zengin G., Bungau S.G., Munteanu M.A., Brisc M.C. (2021). Elucidating the Multi-Targeted Role of Nutraceuticals: A Complementary Therapy to Starve Neurodegenerative Diseases. Int. J. Mol. Sci..

